# Metronidazole; a Potential Novel Addition to the COVID-19 Treatment Regimen

**Published:** 2020-03-30

**Authors:** Reza Gharebaghi, Fatemeh Heidary, Mohammad Moradi, Maryam Parvizi

**Affiliations:** 1 Kish International Campus, University of Tehran, Tehran, Iran.; 2 International Virtual Ophthalmic Research Center (IVORC).; 3 Taleghani Hospital, Ahvaz Jundishapur University of Medical Sciences, Ahvaz, Iran.; 4 Department of Surgery, Iran University of Medical Sciences, Tehran, Iran.; 5 Department of Pathology, Mofid Children’s Hospital, Shahid Beheshti University of Medical Sciences, Tehran, Iran.

**Keywords:** Coronavirus disease, COVID-19, Metronidazole, Cytokines, Interleukins

## Abstract

Coronavirus disease 2019 or COVID-19 has rapidly emerged as a global pandemic. This viral infection involves the upper respiratory tract and could lead to severe pneumonia with respiratory distress or even death. Certain studies have found higher initial plasma levels of most pro-inflammatory cytokines during the course of the infection. In this context, both *in vitro* and *in vivo* studies have revealed that metronidazole could decrease the levels of several cytokines, which are known to increase during the COVID-19 infection, including interleukin (IL)8, IL6, IL1B, tumor necrosis factor (TNF)α, IL12, IL1α, and interferon (IFN)γ, as well as the levels of C-reactive protein (CRP) and neutrophil count.

Furthermore, the drug could decrease neutrophil-generated reactive oxygen species during inflammation. Metronidazole could counteract majority of the immunopathological manifestations of the COVID-19 infection. Therefore, studies with a large sample size are required to determine the efficacy of metronidazole in the treatment of COVID-19 infection.

## Introduction

Coronavirus disease 2019 or COVID-19 has rapidly emerged as a global pandemic since its first report in December 2019 in China (1, 2). The infection involves the upper respiratory tract and could lead to severe pneumonia with respiratory distress or even death (3). Currently, no specific treatment is available, and most strategies are principally symptomatic. Therefore, finding an effective and economical treatment strategy is essential, particularly for those with life-threatening infection (4). Here, we present evidence from the literature of immunological manifestations of the COVID-19 infection and the potential effect of metronidazole in counteracting majority of these immunopathological features.

**Immunopathological evidence of COVID-19**

Following an evaluation of 41 admitted patients with laboratory-confirmed COVID-19 infection in Wuhan, China, Huang et al. found higher initial plasma levels of most pro-inflammatory cytokines, including interleukin (IL)1B, IL1RA, IL7, IL8, IL9, IL10, basic fibroblast growth factor (FGF), granulocyte-colony stimulating factor (GCSF), granulocyte-macrophage colony-stimulating factor (GMCSF), interferon (IFN)γ, IFN-γ inducible protein 10 (IP10), monocyte chemoattractant protein 1 (MCP1), macrophage inflammatory protein (MIP)1A, MIP1B, platelet-derived growth factor (PDGF), tumor necrosis factor (TNF)α, and vascular endothelial growth factor (VEGF) in both intensive care unit (ICU) and non-ICU patients than in the healthy subjects. However, the plasma levels of IL5, IL12p70, IL15, Eotaxin, and RANTES were similar. Further evaluation revealed higher plasma levels of IL2, IL7, IL10, GCSF, IP10, MCP1, MIP1A, and TNFα in ICU-admitted patients than in non-ICU patients (5). 

Chen et al., who retrospectively evaluated 99 patients with laboratory-confirmed COVID-19 infection, reported lymphopenia in 35% of cases, as well as increased levels of neutrophils, IL6, erythrocyte sedimentation rate (ESR), and C-reactive protein (CRP) in 35%, 52%, 85%, and 86% of cases, respectively (6). However, whether the virus directly infects the immune cells, including neutrophils and lymphocytes, is yet to be established (7). 

Zhou et al. conducted a retrospective multicenter study of 191 patients with laboratory-confirmed COVID-19 infection and reported elevated IL6 levels and severe lymphopenia in non-survivors than in the survivors. Univariate analysis of the data revealed significant associations of lymphopenia and elevated IL6 serum levels with mortality (8). 

**Table 1:** Effects of metronidazole on immunopathological manifestations of COVID-19 infection

**COVID-19**	**Metronidazole**
↑IL8 (5)	↓IL8 (10-13)
↑IL6 (6, 8)	↓IL6 (10-15)
↑IL1B (5)	↓IL1B (10-15)
↑TNFα (5)	↓TNFα (11-13, 15-17)
↑CRP (6)	↓CRP (11, 12)
↑IL12 (21)	↓IL12 (11, 13, 14)
↑IFNγ (5)	↓IFNγ (11, 14, 16)
↑Neutrophils (5, 6)	↓Neutrophils (11, 17, 18)
↓Lymphocytes (5, 6, 8)	↑Lymphocytes (11, 17), lymphoproliferative properties (11, 19)

CRP: C-reactive protein; IFN: interferon; IL: interleukin; TNF: tumor necrosis factor.

**Immunopharmacology of metronidazole**

Metronidazole [1-(2-hydroxyethyl)-2-methyl-5-nitroimidazole] and related 5-nitroimidazoles are redox-active prodrugs that act as biocidal agents via their interaction with a nitroreductase homolog (9). Both *in vitro* and *in vivo* studies have revealed that metronidazole decreases the levels of several cytokines, including IL8 (10-13), IL6 (10-15), IL1B (10-15), TNFα (11-13, 15-17), IL12 (11, 13, 14), and IFNγ (11, 14, 16), as well as the levels of CRP (11, 12) and neutrophil count (11, 17, 18). Interestingly, these parameters are shown to increase during the COVID-19 infection (5-7). Moreover, metronidazole could increase the number of circulatory lymphocytes (11, 17) and has lymphoproliferative properties, suggestive of its immunopotentiating effect (11, 19). These immunomodulatory effects of metronidazole have been discussed in detail in a previously published review by Shakir et al. (11). Furthermore, this medication could decrease neutrophil-generated reactive oxygen species during inflammation (11, 20). Table 1 summarizes the effects of metronidazole on the immunopathological manifestations of the COVID-19 infection.

Metronidazole, owing to its immunopharmacological behavior, plays a pivotal role in several essential biological processes. Based on the reported immunological manifestations of COVID-19 infection, it could serve as a potential candidate to counteract majority of the immunopathological features of the disease. Therefore, clinical trials with a large sample size are necessary to determine its efficacy in the treatment of COVID-19 infection. 
